# Treatment of rheumatic immune-related adverse events due to cancer immunotherapy with immune checkpoint inhibitors—is it time for a paradigm shift?

**DOI:** 10.1007/s10067-020-05420-w

**Published:** 2020-09-28

**Authors:** Katerina Chatzidionysiou, Matina Liapi, Georgios Tsakonas, Iva Gunnarsson, Anca Catrina

**Affiliations:** 1 Rheumatology Unit, Karolinska University Hospital, Stockholm, Sweden; 2 Department of Medicine Solna, Karolinska Institute, Stockholm, Sweden; 3 Thoracic Oncology Center, Karolinska University Hospital, Stockholm, Sweden; 4 Department of Oncology and Pathology, Karolinska Institute, Stockholm, Sweden

**Keywords:** Cancer immunotherapy, Immune checkpoint inhibitors, Immune-related adverse events, Therapy

## Abstract

Immunotherapy has revolutionized cancer treatment during the last years. Several monoclonal antibodies that are specific for regulatory checkpoint molecules, that is, immune checkpoint inhibitors (ICIs), have been approved and are currently in use for various types of cancer in different lines of treatment. Cancer immunotherapy aims for enhancing the immune response against cancer cells. Despite their high efficacy, ICIs are associated to a new spectrum of adverse events of autoimmune origin, often referred to as immune-related adverse events (irAEs), which limit the utility of these drugs. These irAEs are quite common and can affect almost every organ. The grade of toxicity varies from very mild to life-threatening. The pathophysiological mechanisms behind these events are not fully understood. In this review, we will summarize current evidence specifically regarding the rheumatic irAEs and we will focus on current and future treatment strategies. Treatment guidelines largely support the use of glucocorticoids as first-line therapy, when symptomatic therapy is not efficient, and for more persistent and/or moderate/severe degree of inflammation. Targeted therapies are higher up in the treatment pyramid, after inadequate response to glucocorticoids and conventional, broad immunosuppressive agents, and for severe forms of irAEs. However, preclinical data provide evidence that raise concerns regarding the potential risk of impaired antitumoral effect. This potential risk of glucocorticoids, together with the high efficacy and potential synergistic effect of newer, targeted immunomodulation, such as tumor necrosis factor and interleukin-6 blockade, could support a paradigm shift, where more targeted treatments are considered earlier in the treatment sequence.

## Introduction

The pivotal role of the immune system in cancer and the concept of cancer immunotherapy is far from new [1]. It has been known for long that immune surveillance is responsible for elimination of cancer cells in the very initial stages of carcinogenesis [2]. Creation of neo-antigens, foreign antigens on the surface of cancer cells, which are the result of genetic and epigenetic changes, makes cancer cells a detectable target for destruction by the immune system. However, cancer cells can develop survival mechanisms and escape immune detection and destruction through induction of tolerance among tumor-specific T cells and inhibition of T cell functions within the tumor microenvironment [3].

Several different approaches of immunotherapy in cancer have been or are currently being developed. Immune checkpoint inhibitors (ICIs) restore the immune response against tumors. Vaccination with tumor antigens activates effector immune cells to tack neoplastic cells, albeit this strategy has not been so successful [4]. Adoptive cellular therapy with administration of immune cells directly to patients, administration of oncolytic viruses for initiating systemic antitumor activity and ways of supplying co-stimulatory signals to enhance T cell activity, such as with cytokine administration in order to stimulate the host’s immune system (IL2, IFN-α), are some other examples of cancer immunotherapy [2]. Chimeric antigen receptor (CAR) T cell therapy is another promising immunotherapy using gene transfer technology to induce a patient's cytotoxic T lymphocytes to express CARs stably [5].

## Immune checkpoints

Among these different strategies, the most widely used today is the blockade of some specific immune checkpoints, such as the cytotoxic T lymphocyte–associated antigen 4 (CTLA-4) and programmed death 1 (PD-1)/programmed death ligand 1 (PD-L1) acting as negative regulators of T cell immune function, using the so called CPIs. During recent years several monoclonal antibodies to inhibit these targets have been developed and approved for the treatment of melanoma, non-small cell lung cancer, and other cancers. Other inhibitory immune checkpoints exist, such as lymphocyte activation gene 3 (LAG-3), T cell immunoglobulin, and mucin domain 3 (TIM-3), V-domain immunoglobulin suppressor of T cell activation (VISTA). We are going to focus on PD-1/PD-L1 and CTLA-4 since there are approved treatments targeting them.

**Fig. 1:**
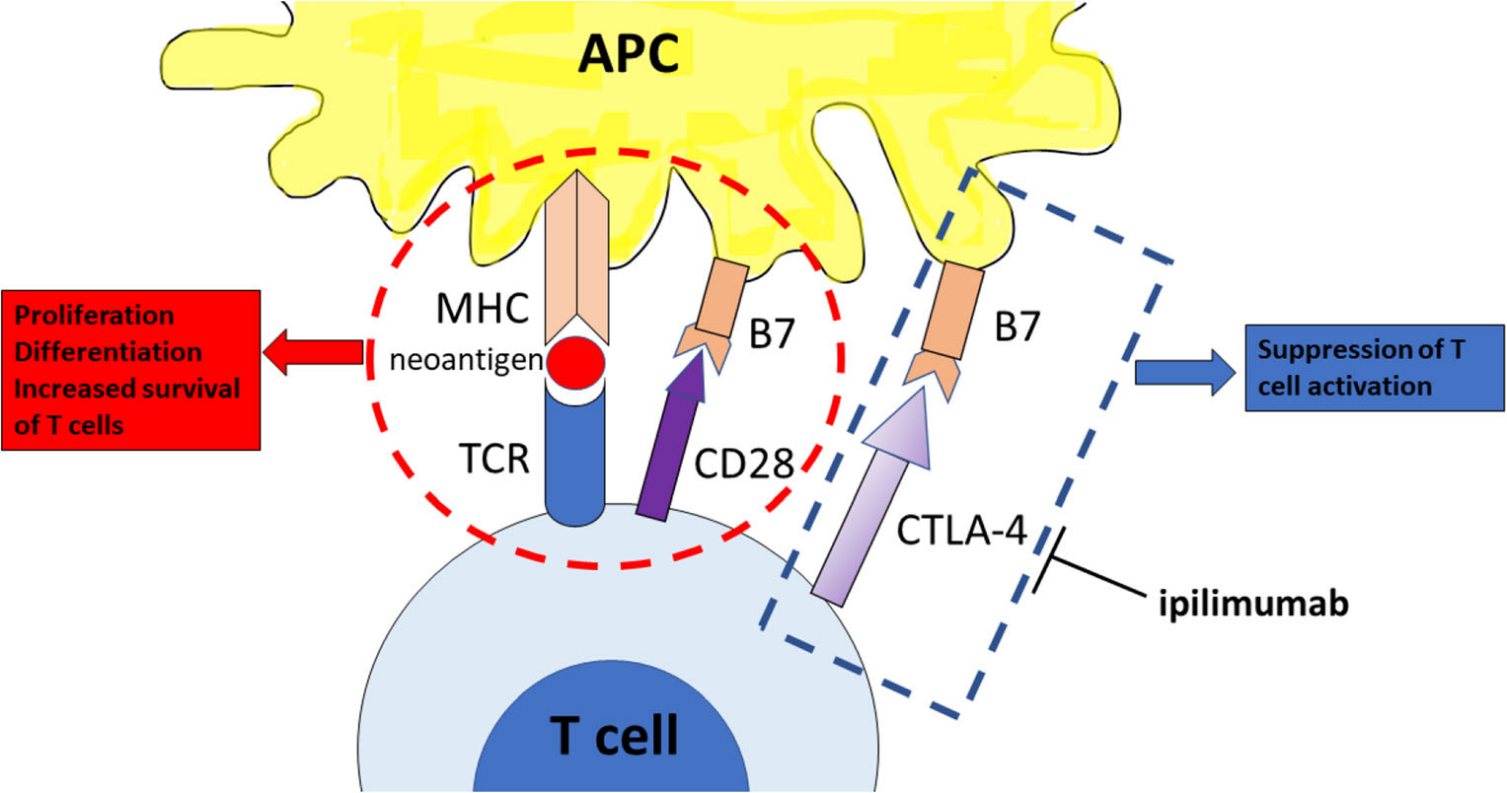
The CTLA-4 immune checkpoint

T cells require more than one stimulatory signal in order to be activated. Binding of B7-1 (CD80) or B7-2 (CD86) molecules on the antigen-presenting cells with CD28 on the T cells gives the co-stimulatory signal required for the activation of T cell, after the binding of T cell receptor (TCR) to MHC [6]. This leads to proliferation, differentiation and increased survival of T cells. CTLA-4 is a CD28 homolog with higher affinity to B7, but unlike CD28, it does not produce a stimulatory signal leading to suppression of T cell activation [7] (Fig. 1). The relative amount of CD28:B7 binding versus CTLA-4:B7 binding determines whether a T cell will undergo activation or will become anergic [8]. Stimulatory signals induce upregulation of CTLA-4 [9]. In some cases however, such as in regulatory T cells, CTLA-4 is constitutively expressed and significantly contributes to their immune suppressive functions [10].

**Fig. 2:**
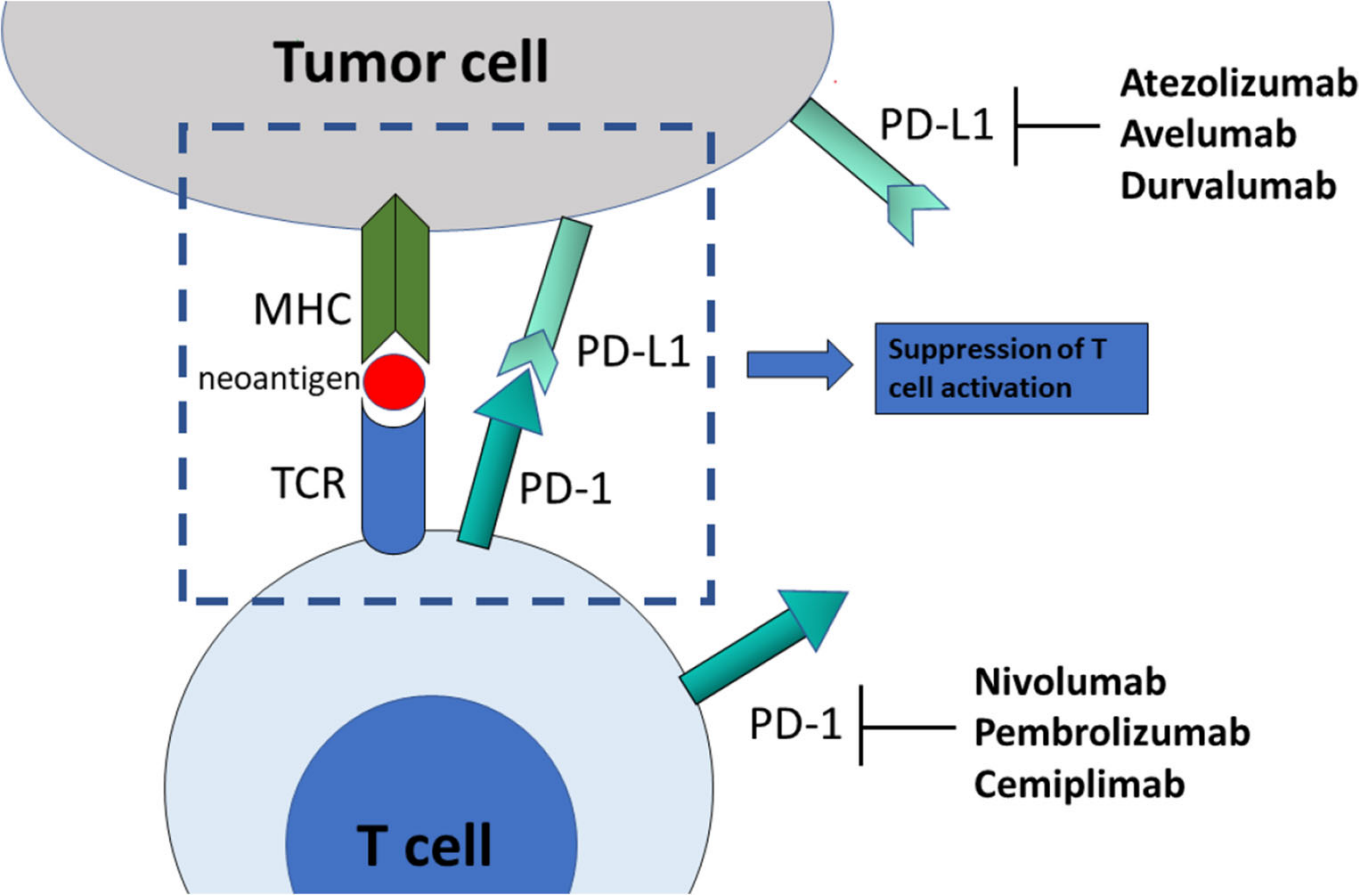
The PD-1 immune checkpoint

Similarly to CTLA-4, PD-1, a member of the B7 family of co-stimulatory receptors, inhibits T cell activation, proliferation and survival through binding to PD-L1 and PD-L2 [11] (Fig. 2). Although both PD-L1 and PD-L2 are ligands of PD-1 and both downregulate T cell effector function, these two ligands differ in several aspects. PD-L1 is constitutively expressed at low levels and is induced on nearly all tissues upon interferon-gamma signaling. In contrast, PD-L2 expression is restricted mainly to antigen-presenting cells. PD-L2 has a much stronger affinity for PD-1 than does PD-L1[12]. There is a difference in the expression of these two ligands in different tumor types, something which might have therapeutic implications [13]. PD-1 is also a marker of “exhausted” T cells. T cell exhaustion is a state of hypofunctional T cells in response to chronic antigen load, such as during chronic infections and cancer, resulting in suboptimal control of infections and tumors [14]. However, more recently, the definition of T cell exhaustion has been altered. It is now known that exhausted T cells is a heterogeneous group including T cells that retain some effector function and play a crucial role in limiting infections and tumor growth [15].

Inhibition of the above immune checkpoints leads to activation and proliferation of a higher number of effector T cells and boosting the antitumor response. The exact mechanisms however remain to be fully elucidated. In more detail, CTLA-4 blockade leads to activation and proliferation of more T cell clones and reduces T regulatory cell mediated immunosuppression [6]. PD-1 blockade restores the activity of antitumor T cells that have become quiescent [6]. PD-L1 and PD-L2 are more widely expressed compared with the B7 ligands for CTLA-4. Inhibiting PD-L1 specifically, as opposed to PD-1 inhibition, will block PD-1:PD-L1 interactions while preserving PD-1:PD-L2 interactions, since PD-1 has a higher affinity to PD-L2 than PD-L1 [16]. That might have potential implications for a more favorable toxicity profile of PD-L1 targeted inhibition.

Hitherto, seven CPIs have been approved by regulatory authorities for the treatment of various solid tumors and hematological malignancies, either alone or in combination, and for different stages of the diseases [17]. In Table 1, the approved CPIs, their indication, target molecule and route of administration is summarized.

**Table 1:** Approved immune checkpoint inhibitors, their targets, and indications

Monoclonal antibody	Target	Indications
Ipilimumab	CTLA-4	Advanced renal cell carcinoma, metastatic colorectal cancer, cutaneous melanoma, unresectable or metastatic melanoma
Nivolumab	PD1	Metastatic small cell lung cancer, unresectable or metastatic melanoma, locally advanced or metastatic urothelial carcinoma, metastatic colorectal cancer, hepatocellular carcinoma, metastatic non-small cell lunch cancer, advanced renal cell carcinoma, classical Hodgkin lymphoma, recurrent or metastatic squamous cell carcinoma of the head and neck
Pembrolizumab	PD1	Melanoma, non-small cell lung cancer, head and neck squamous cell cancer, Hodgkin lymphoma, Merkel cell carcinoma, hepatocellular carcinoma, gastric cancer, urothelial carcinoma, cervical cancer
Cemiplimab	PD1	Metastatic and locally advanced cutaneous squamous cell carcinoma
Atezolizumab	PD-L1	Urothelial carcinoma, metastatic non-small cell lung cancer
Avelumab	PD-L1	Metastatic Merkel cell carcinoma, locally advanced or metastatic urothelial carcinoma
Durvalumab	PD-L1	Unresectable stage III non-small cell lung cancer, locally advanced or metastatic urothelial carcinoma

## Immune-related adverse events—a new category of toxicity

Since immunological checkpoints are important regulators of the immune system, contributing to self-tolerance, inhibition of these pathways leads not unexpectedly to overactivation of the immune system and genesis of autoimmune phenomena. Their exact pathophysiology is not yet entirely understood. This new category of toxicities, the immune-related adverse events (irAEs), is remarkably common, with approximately 50% of patients treated with CPI experiencing some form of irAE [18]. They vary significantly in their severity, ranging from very mild to life-threatening. They can affect any organ system, either a single one or multiple organs simultaneously [19]. Patrick Arnaud-Coffin et al. in a systematic review of CPI irAEs in RCTs reported grade ≥ 3 AEs for 14% of patients treated with PD(L)-1 inhibitors, 34% of patients treated with CTLA-4 inhibitors, 55% of patients on CPI combinations and 46 % of patients on immunotherapy-chemotherapy combination [20]. The profile of irAEs was different among the treatment categories. The use of CPI, especially in combination, is associated with significant rates of grade ≥ 3 AEs. Colitis, dermatitis, pneumonitis, and hypophysitis are some examples of irAEs. Interestingly, development of an irAEs is usually associated with better response to treatment [21].

Among these irAEs, rheumatic complications, such as arthritis, myositis, sicca syndrome, and polymyalgia rheumatica, are relatively common and challenging not only to diagnose but also to treat. Rheumatologic irAEs can occur and persist long after the cessation of the CPI treatment in contrast to all others non-rheumatic irAEs [22].

The true frequency of rheumatic irAEs is not well characterized mainly because of underreporting, partly due to lack of severity leading to hospitalization or death but also because they can occur as a late as 2 years after the CPI treatment. In addition, patients with pre-existing rheumatic conditions are excluded from the clinical trials due to possible exacerbation of autoimmune toxicity. Thus, the frequency estimates for rheumatic irAEs vary substantially. For arthralgia/arthritis the frequency reported ranges from 1 to 43% and for myalgia including polymyalgia rheumatica (PMR) like syndrome from 2 to 20%. Sicca syndrome is been reported in 5% of patients receiving monotherapy and 10% of those receiving combination therapy [23]. More than half of the vasculitis cases associated with cancer immunotherapies are related to CPIs, and increased cases of granulomatous disorders (sarcoidosis), systemic sclerosis, lupus, antiphospholipid syndrome, and eosinophilic fasciitis are reported [23].

Acquiring a deeper understanding of the biology of immune checkpoints and their inhibition is crucial in order to approach more optimal management strategies. Several recommendations regarding the management of irAEs have been published to date [24–27].

## Management of rheumatic irAE

There are some basic principles regarding treatment approach of irAEs that we need to acknowledge. The therapeutic decisions should be taken after discussion between rheumatologists, oncologists, and patients. Continuation or discontinuation of the CPI should be guided by the severity of the irAE, the effectiveness of the CPI and the immunosuppressive treatment, making the interdisciplinary approach crucial for the optimal management of the patients. Additionally, we should always keep in mind that we aim at a fine balance, where the degree of immunosuppression should be high enough to control the irAE but at the same time preserve the antitumoral immune response.

According to recently published points to consider from the European League against Rheumatism (EULAR), symptomatic treatment including non-steroidal anti-inflammatory drugs and/or analgesics should be the initial treatment for mild-to-moderate rheumatic irAE [25]. Local treatment with intra-articular glucocorticoids can be considered in case of monoarthritis or oligoarthritis, with or without combination with symptomatic treatment. If this is insufficient and tissue inflammation is still evident, systemic glucocorticoids should be considered. Some of the rheumatic irAEs might require high doses of glucocorticoids, and case reports and case series have shown that even arthritis, which normally respond to low–medium doses in most cases, might need high doses of glucocorticoids in the treatment of irAE [28]. Identification of non-steroid treatment modalities in rheumatic irAE is thus an issue of concern in severe cases.

## Glucocorticoids—friend or foe?

As described above, systemic glucocorticoids are quite high in the treatment pyramid for the control of inflammation seen in irAEs. However, there is limited evidence regarding the safety of glucocorticoids, especially in high doses, in terms of a potential negative impact on anti-tumoral responses. Glucocorticoids have been negatively associated with prognostic feature of immunotherapy, in particular for cancers outside of the central nervous system [29]. It has also been shown in mouse models that both endogenous and exogenous glucocorticoids can inhibit anticancer immune responses [30]. One study tested in vitro the influence of clinically relevant doses of dexamethasone and an anti-TNF monoclonal antibody [31]. In this study, even low doses of corticosteroids markedly impaired the anti-tumor activity of tumor-infiltrating lymphocytes. In contrast, a standard clinical dose of infliximab, a chimeric anti-tumor necrosis factor (anti-TNF) monoclonal antibody, only had a minor effect on T cell activation and tumor-killing. The activity of lymphocytes was restored after withdrawal of steroids. Exogenous glucocorticoids used at clinically relevant concentrations also have immunosuppressive effects on the capacity of dendritic cell to present tumor antigens as well as on T cell activation and tumor-killing activity [32]. These data could suggest that steroid-sparing strategies and early initiation of anti-TNF therapy should be considered for the treatment of irAEs in immuno-oncology.

Despite these in vitro effects, clinical data on the use of corticosteroids have been contradictory, though only few and small reports are currently available. In a case series with 36 patients with pre-existing rheumatic disease before anti-PD1 therapy or de novo rheumatic irAEs on anti-PD1 therapy, the majority of patients (30 out of 36) received glucocorticoids [33]. High response rates to anti-PD1 treatment were still observed, especially in the melanoma group. No overt differences were observed in the duration of corticosteroids in patients who exhibited ongoing responses compared to those with primary or acquired resistance. In a systematic literature review, the data suggested the concomitant administration of corticosteroids and immune checkpoint inhibitors may not necessarily lead to poorer clinical outcomes [34].

However, it is important to note that steroid administration at doses higher than 10 mg prednisolone equivalent were part of the exclusion criteria in the clinical trials that led to the approval of ICIs in non-small-cell lung cancer [35–37]. Retrospective data in non-small-cell lung cancer patients treated with ICIs have demonstrated worse response rates, progression-free survival and overall survival with steroid administration at doses higher than 10 mg of prednisolone equivalent at treatment initiation [38]. A retrospective study in NSCLC patients receiving steroids for palliation of cancer-related symptoms showed worse efficacy of concomitant ICIs [39]. High-dose steroid administration (≥ 1 mg/kg/day) is the main treatment option for the management of severe grade III–IV irAEs, although data on the clinical outcome of patients who received steroids due to the development of irAEs derive mostly from melanoma studies. These have reported that their administration does not influence ICI efficacy [40].

To conclude, there is at least a theoretical background suggesting a negative impact of long-term use of corticosteroids on the duration of anti-tumor response, but large, prospective studies are needed.

## Targeted treatment—time for a paradigm shift?

According to the recently published points to consider, in case of active rheumatic irAE requiring dose of glucocorticoids higher than 10 mg/day of equivalent prednisone, a conventional synthetic DMARD (csDMARD), such as methotrexate, hydroxychloroquine or sulfasalazine, should first be considered [25]. There are several case reports and case series demonstrating efficacy and safety of these drugs for the management of rheumatic irAEs [25]. In case of severe irAE or after inadequate response to csDMARDs, a bDMARD may be considered. During the last two decades the therapeutic armamentarium for various rheumatic diseases has been broadened dramatically with the advent of selective immune-targeted therapeutics based upon pathogenesis driven principles. Numerous monoclonal antibodies and receptors targeting pro-inflammatory key cytokines (such as IL6, IL17, IL1, TNF) and immune cells (such as B cells) have been proven effective and with an acceptable safety profile for chronic rheumatic conditions. These drugs consist a large group called biologic disease-modifying anti-rheumatic drug (bDMARDs) (Table 2).

**Table 2:** Approved biological and targeted synthetic disease-modifying anti-rheumatic drugs used for the treatment of various rheumatic conditions

	Target	Indications
Biological DMARDs (bDMARDs)		
Infliximab	TNF	RA, PsA, axSpA
Adalimumab	TNF	RA, PsA, axSpA
Etanercept	TNF	RA, PsA, axSpA
Certolizumab pegol	TNF	RA, PsA, axSpA
Golimumab	TNF	RA, PsA, axSpA
Sekukinumab	IL-17	PsA, axSpA
Tocilizumab	IL-6R	RA, GCA
Sarilumab	IL-6	RA
Abatacept	T cell co-stimulation	RA, PsA
Rituximab	CD-20 (B cells)	RA, AAV, SLE*
Belimumab	BLyS	SLE
Anakinra	IL-1	RA**, still’s disease, periodical fever syndromes
Targeted synthetic DMARDs (tsDMARDs)		
tofacitinib	JAK1/JAK3	RA, PsA
baricitinib	JAK1/JAK2	RA
upadacitinib	JAK1	RA

*TNF*, tumor necrosis factor; *IL*, interleukin; *BLyS*, B-lymphocyte stimulator; *JAK*, Janus kinase; *RA*, rheumatoid arthritis; *PsA*, psoriatic arthritis; *axSpA*, axial spondyloarthritis; *GCA*, giant cell arteritis; *AAV*, ANCA (anti-neutrophil cytoplasmic antibodies)-associated vasculitis; *SLE,* systemic lupus erythematosus

*Off-label use

**Approved but not routinely used due to limited efficacy compared with other bDMARDs

Especially for arthritis cases, tumor necrosis factor (TNF) and interleukin-6 (IL6) blockade have been successfully used. These two cytokines play a central role in the immunopathogenesis of rheumatoid arthritis. TNF, a critical cytokine for both physiological and pathological processes, has a central role in the pathogenesis of many inflammatory disorders, such as rheumatoid arthritis and Crohn’s disease. It is also a key mediator of cancer associated inflammation [41]. There are several drugs that target TNF available today with well-established efficacy and effectiveness as well as a good short- and long-term safety profile [42]. TNF inhibition has been used with success in cases of severe colitis and arthritis [43]. For severe CPI induced colitis, we have witnessed a treatment paradigm shift the last years, with TNF blockade being the first-line treatment, and even as a prophylactic measure [44, 45]. Concurrent administration of ICIs with infliximab is currently under investigation in the TICIMEL trial (NCT03293784). For arthritis, use of TNF inhibitors is considered after the failure of first-line treatments with GCs and csDMARDs, and for severe cases.

IL-6 is a key cytokine in rheumatoid arthritis (RA). It is secreted from a wide variety of cells including macrophages, T cells, B cells, and synovial fibroblasts, and is regarded as upper-rank cytokine in the hierarchical cytokine network involved in the pathogenesis of RA. It has a wide range of functions, such as in B cell proliferation and antibody production, hematopoiesis, and T cell differentiation [46, 47]. Tocilizumab is a humanized monoclonal antibody against IL-6 receptor, approved for the treatment of active RA both as monotherapy and in combination with methotrexate. Its efficacy and acceptable safety profile has been demonstrated in several large randomized controlled trials [48–51]. In a study on 87 patients who developed irAEs after treatment with nivolumab, clinical improvement was observed in 27 of 34 patients who received tocilizumab [52]. In a small case series tocilizumab was used successfully for the treatment of severe polyarthritis induced by CPI [53].

As in the case of glucocorticoids, a major concern with potent-targeted treatments is the risk of attenuation of the anti-tumoral effect of the CPI, especially with long duration of treatment. In contrast to other irAEs, such as colitis, that can subside after a single administration of a biologic agent (such as an anti-TNF monoclonal antibody), rheumatic irAEs tend to be more chronic and require long-term immunomodulation for optimal control of the inflammation. A recent study with a median follow-up of 9 months reported that anti-tumor responses were not adversely affected in patients treated with TNF inhibitors [54]. As discussed above, there is evidence from preclinical studies to support the superiority of targeted versus broader immunomodulation (anti-cytokine therapy vs. glucocorticoids), with the first having only a minor influence on T cells. In addition to that, there seems to be a potential synergistic effect of TNF inhibitors with CPI [55, 56]. Treating mice with TNF inhibitors concomitantly with combined CTLA-4 and PD-1 immunotherapy ameliorated colitis and, in addition, improved anti-tumor efficacy. Combined blockade of IL6 and PD1/PD-L1 signaling was also found to enhance tumor-specific Th1 responses and subsequent anti-tumor effects in tumor-bearing mice [57].

Data regarding other bDMARDs, such as secukinumab, an anti-IL17 monoclonal antibody and rituximab, an anti-CD20 monoclonal antibody are limited. A particular situation is related to abatacept, a recombinant fusion protein comprising the extracellular domain of human CTLA-4 and a fragment of the Fc domain of human IgG1, used for treatment of RA. It acts through inhibition of the CD-28-B7-mediated T cell co-stimulation at the level of dendritic cells and thus abrogate T cell co-stimulation upstream of CTLA-4 and PD-1/PL-L1 pathways, potentially leading to a rapid and global cell energy. Taking into account the structure and mechanism of action of abatacept, one could consider its use in life-threatening events, but this remains to be proven. Indeed, a case report showed successful remission of nivolumab-induced myocarditis [58]. Its efficacy and safety remains to be proven in studies.

During the last years, a new category of disease-modifying anti-rheumatic drugs (DMARDs) has appeared, the so called targeted synthetic DMARDs (tsDMARDs), consisting of the Janus Kinase (JAK) inhibitors (Table 2). The JAK family comprises four members: JAK1, JAK2, JAK3, and TYK2. They are cytoplasmic tyrosine kinases that mediate the intracellular signaling by association with type 1 and type II cytokine receptors [59]. JAK activation leads to activation of their downstream substrates, the signal transducer and activator of transcription (STAT) proteins, followed by their nuclear translocation and subsequent activation of target genes [60]. IL-6 is one of the cytokines that use the JAK/STAT pathway to exert their intracellular signal. Additionally, type I interferons signal through JAK1 and JAK3, while type II interferon (INFγ) signals through JAK2 [61]. IFNγ is essential for PD-L1 and PD-L2 expression and is a marker of response to CPI [62]. Since the JAK/STAT pathway is related to INF signaling, it is logical to hypothesize that JAK inhibition might have a place in the management of CPI-related adverse events but also resistance to therapy. Indeed, preclinical studies have shown that combination of JAK inhibition and CPI enables to overcome resistance to CPI possibly by reducing inflammation in the tumor microenvironment [63]. As INFγ is not a desirable target in the context of cancer immunotherapy due to its importance for PD-L expression, JAK2-INFγ should be retained. The first generation of JAK inhibitors, such as tofacitinib and baricitinib affect JAK2 (baricitinib more than tofacitinib), but the second generation of JAK inhibitors are more selective, such as upadacitinib, a selective JAK1 inhibitor recently approved for the treatment of RA. The clinical efficacy and safety of JAK inhibitors in the context of irAE has yet to be proven.

## Conclusion

**Fig. 3:**
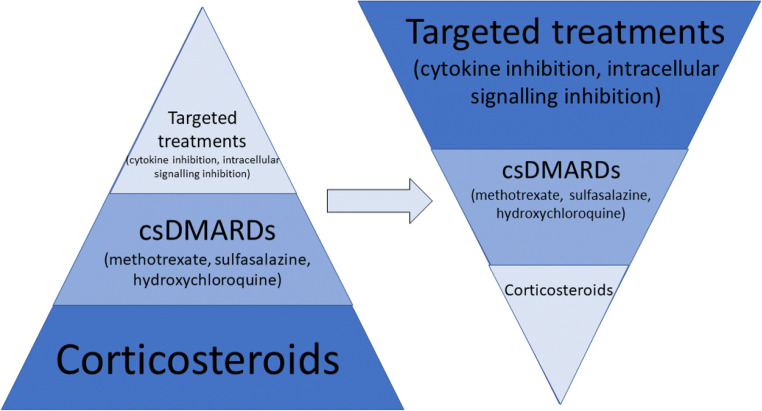
Paradigm shift of treatment approach of rheumatic irAEs with CPI

Better understanding of the pathophysiology of the rheumatic irAEs is needed, aside with predictors of development of this new type of AEs. Treatment guidelines largely support the use of glucocorticoids as first-line therapy, when symptomatic therapy is not efficient, and for more persistent and/or moderate/severe degree of inflammation. Targeted therapies are higher up in the treatment pyramid, after inadequate response to glucocorticoids and conventional, broad immunosuppressive agents, and for severe forms of irAEs. However, preclinical data provide evidence that raise concerns regarding the potential risk of impaired anti-tumoral effect, with some clinical evidence regarding the negative effect of corticosteroid treatment on the efficacy of ICIs in non-small-cell lung cancer. This important risk of glucocorticoids, together with the high efficacy and potential synergistic effect of newer, targeted immunomodulation, such as TNF and IL6 blockade, support a paradigm shift and an invert treatment pyramid, where csDMARDs and bDMARDs should be considered earlier in the treatment sequence (Fig. 3). Future data from prospective studies and randomized clinical trials, some of them ongoing, will provide more evidence regarding this highly relevant clinical question.
